# Space-time patterns and associated factors of leishmaniasis in Colombia, 2008-2016

**DOI:** 10.15446/rsap.V25n4.97936

**Published:** 2023-07-01

**Authors:** Elena M. Hurtado, Camila S. Fonseca de Oliveira, Marcelo Teixeira Pava, Mariana Olímpia Kòhler Marra Morato, David Soeiro Barbosa, Danielle Ferreira de Magalhães Soares

**Affiliations:** 1 EM: B. S. Veterinary Medicine and Zootechnics. M. Sc. Animal Science. Veterinary School, Federal University of Minas Gerais (UFMG). Belo Horizonte/MG, Brazil. solel989@gmail.com Federal University of Minas Gerais Veterinary School Federal University of Minas Gerais Belo Horizonte MG Brazil; 2 CF: B. S. Veterinary Medicine. M. Sc. Animal Science. Ph. D. Animal Science. Veterinary School, Federal University of Minas Gerais (UFMG). Belo Horizonte/MG, Brazil. sfo.camila@gmail.com Federal University of Minas Gerais Veterinary School Federal University of Minas Gerais Belo Horizonte MG Brazil; 3 MT: B. S. Veterinary Medicine. Grad. Student Animal Science with an emphasis on Epidemiology. Spec. Public Health with an emphasis on zoonoses and population control of dogs and cats. Veterinary School, Federal University of Minas Gerais (UFMG). Belo Horizonte/MG, Brazil. marcelo_thelin@hotmail.com Federal University of Minas Gerais Veterinary School Federal University of Minas Gerais Belo Horizonte MG Brazil; 4 MK: B. S. Veterinary Medicine and Zootechnics. M. Sc. Animal Science. Veterinary School, Federal University of Minas Gerais (UFMG). Belo Horizonte/MG, Brazil. marykohler@gmail.com Federal University of Minas Gerais Veterinary School Federal University of Minas Gerais Belo Horizonte MG Brazil; 5 DS: B. S. Veterinary Medicine. M. Sc. Epidemiology in Public Health. Ph. D. Epidemiology in Public Health. Institute of Biological Sciences, Federal University of Minas Gerais (UFMG). Belo Horizonte/MG, Brazil. davidsoeiro@gmail.com Institute of Biological Sciences Institute of Biological Sciences Federal University of Minas Gerais Belo Horizonte MG Brazil; 6 DF: B. S. Veterinary Medicine. M. Sc. Animal Science. Ph. D. Animal Science. Veterinary School, Federal University of Minas Gerais (UFMG). Belo Horizonte/MG, Brazil. danifml@yahoo.com.br Federal University of Minas Gerais Veterinary School Federal University of Minas Gerais Belo Horizonte MG Brazil

**Keywords:** Visceral leishmaniasis, cutaneous leishmaniasis, Colombia, epidemiology *(source: MeSH, NLM)*, Leishmaniasis visceral, leishmaniasis cutánea, Colombia, epidemiología *(fuente: DeCS, BIREME)*

## Abstract

**Objective:**

To analyze the space-time aspects and epidemiological characteristics of leishmaniasis in Colombia from 2008 to 2016.

**Methodology:**

This was an cross-sectional and ecological study of the morbidity and mortality from leishmaniasis and its spatial, temporal, and socio-demographic associated factors based on reported cases of ACL and VL obtained from the National Public Health Surveillance System (Sivigila) of the Ministry of Health of Colombia.

**Results:**

In all, 99,503 cases of leishmaniasis were reported in Colombia in the 9-year period (2008-2016), where ACL accounted for 99.78% of the cases, with 14 deaths, and VL accounted for 0.21% of the cases, with four deaths. People living in the rural areas were the most affected by both forms of leishmaniasis. In the spatial analysis, ACL was widely distributed in Colombian territory, but the patterns of occurrence were not consistent throughout the study period. Further, the majority of the population affec-ted by VL resided in the Caribbean and Central regions, during the second three-year study period, indicating dispersion of the disease.

**Conclusions:**

The recent epidemiological patterns of leishmaniasis show variations in morbidity and mortality, with a higher incidence of ACL than of VL and high concentration of cases in certain regions of Colombia. This study contributes to a better unders-tanding of this important public health problem so that measures to control the spread of this disease in Colombia can be intensified.

## RESUMEN

**Objetivo:**

Analizar los aspectos espaciotemporales y las características epidemiológicas de la leishmaniasis en Colombia de 2008 a 2016.

**Metodología:**

Se trata de un estudio transversal y ecológico de la morbilidad y mortalidad por leishmaniasis y sus factores espaciales, temporales y sociodemográficos asociados, con base en casos reportados de LCA y LV obtenidos del Sistema Nacional de Vigilancia en Salud Pública (Sivigila) del Ministerio de Salud de Colombia.

**Resultados:**

En total, se notificaron 99 503 casos de leishmaniasis en Colombia en el periodo de 9 años (2008-2016), donde el LCA representó el 99,78% de los casos, con 14 defunciones, y la LV representó el 0,21% de los casos, con cuatro fallecidos. Las personas que viven en las zonas rurales fueron las más afectadas por ambas formas de leishmaniasis. En el análisis espacial, el LCA se distribuyó ampliamente en el territorio colombiano, pero los patrones de ocurrencia no fueron consistentes a lo largo del periodo de estudio. Además, la mayoría de la población afectada por LV residió en las regiones del Caribe y Centro, durante el segundo periodo de estudio de tres años, lo que indica la dispersión de la enfermedad.

**Conclusiones:**

Los patrones epidemiológicos recientes de la leishmaniasis muestran variaciones en la morbimortali-dad, con mayor incidencia de LCA que de LV y alta concentración de casos en algunas regiones de Colombia. Este estudio contribuye a una mejor comprensión de este importante problema de salud pública para que se puedan intensificar las medidas para controlar la propagación de esta enfermedad en Colombia.

Leishmaniasis is a neglected tropical zoonotic disease with worldwide distribution, presenting high disease burden in Latin American countries. The two main forms of presentation are American cutaneous leishmaniasis (ACL) - endemic in 18 countries of the Americas, affecting the skin and mucous membranes -and visceral leishmaniasis (VL) - endemic in 12 countries, with greater severity than ACL, as it affects the liver and spleen 1.

Colombia is one of the three main countries in the world with the largest number of Leishmania species in its territory, and the disease of wild origin is predominant 2. The reservoirs of the pathogen are mammals belonging to the orders Rodentia, Didelphimorphia, Carnivora, Pilosa, Lagomorpha, and Perissodactyla 3,4. In the case of VL, dogs are the main domestic reservoirs - a sentinel for human infection; once infected, humans present clinical manifestations of the disease and may die from visceral involvement if the disease is not diagnosed and treated in time 3,5-11.

Leishmaniasis has been frequently reported since 1983, with epidemic outbreaks of cutaneous leishmaniasis (CL) being reported since 1984, and in recent years, its spread in urban areas has been observed in Colombian territory. The first case of CL was reported in 1944, with an outbreak of CL in the northern coast 2.

The governing body responsible for the notification and analysis of events affecting public health in Colombia is the National Public Health Surveillance System (Sivigila), created and regulated by Decree No. 3518 of 2006. According to the "Protocol for Public Health Surveillance of Leishmaniasis," confirmed cases of all forms of leishmaniasis must be notified individually on a weekly basis and suspected cases of VL must be notified immediately. In addition, every patient over 18 years of age with a confirmed diagnosis of VL must undergo a laboratory test for the human immunodeficiency virus (HIV) in order to detect the occurrence of co-infection 3. This study aimed to analyze the spatial-temporal and socio-demo-graphic associated factors of leishmaniasis in Colombia during the years 2008 to 2016.

## METHODOLOGY

### Study Area

Colombia covers a territorial area of 1,141,748 km^2^ and is the third most populous country in Latin America after Mexico and Brazil, with an estimated population of 48,258,494 inhabitants and a population density of 43 inhabitants/km2 12. It has a tropical climate, with variations in altitude, temperature, humidity, wind, and rainfall according to the geographical region 13,14.

**Figure 1:**
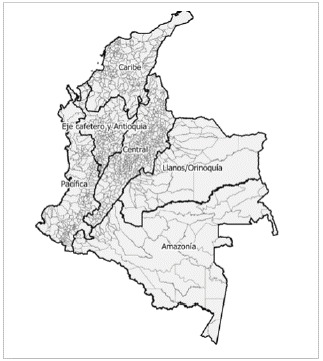
Map of the regions of Colombia

Its territory is subdivided by the National Administrative Department of Statistics (DANE) into 32 departments and 1121 municipalities. The country is classified into six natural regions based on relief, ecosystem, and climate: Caribbean, Eje Cafetero y Antioquia, Pacífica, Central, Llanos/Orinoquia, and Amazon (Figure 1).

Regions of the country: 1- Caribbean; 2- Eje Cafetero y Antioquia; 3- Pacific; 4- Central; 5- Llanos/Orinoquía, and 6- Amazon. Source: DANE, 2018

### Study design

A cross-sectional and ecological study were conducted to analyze the morbidity and mortality from leishmaniasis and associated socio-demographic factors, with a focus on the spatial and temporal aspects of the reported cases of ACL and VL obtained from the National Public Health Surveillance System - Sivigila of the Colombian Ministry of Health.

### Data analysis

#### Indicators

The annual incidence rates were calculated using population data from the 2012 population census and the estimated population for the other years was obtained from the National Administrative Department of Statistics (DANE). The analyses were carried out using the Stata statistical package version 14.0.

### Associated factors

Gender, age, ethnicity, and housing area were the variables analyzed to compare the risk factors for VL and ACL, and a multiple forward logistic regression was carried out 15. Initially, the variables were included one-by-one and the factors with a tendency to significance (p < 0,20) were considered for inclusion in the multivariate analysis. Then, only the variables with p <0,05 were used for the likelihood ratio test, which had the type of leishmaniasis (ACL or VL) as the dependent variable. As independent variables, gender (female and male), age (under 5, 5-9, 10-20, 21-50, and over 50 years), ethnicity (indigenous, Romani/gypsy, black/ mulatto/Afro-Colombian, palenquero, and raizal), and housing area (rural or urban) were considered.

The adjustment of the final logistic regression model was verified with the evaluation of the standard deviation 16, and the results were presented as odds ratio (OR) with a 95% confidence interval (CI).

### Spatial and temporal analysis

Time series and thematic maps of the Colombian territory were prepared, taking into account the number of reported (probable and confirmed) cases per ACL and VL site for each municipality. PAHO'S division by the natural break method was used, and such intervals were calculated by the QGÍS software version 2.18. The minimum and maximum values were considered, which were standardized for the study period.

A matrix of neighborhoods of municipalities and subsequent identification of clusters of areas with high risk of leishmaniasis was created. Since the islands of San Andrés, Providencia, and Santa Catalina are geographically separated from the map, they were excluded to avoid interference in the spatial analyses.

The global Moran index (Moran I) was used to verify the occurrence of positive or negative spatial autocorrelation. Local Indicator of Spatial Association (LISA) were used to identify spatial clusters. The results are presented in four quadrants: Quadrant 1 - High/High (positive values and positive means) corresponding to areas of higher priority, Quadrant 2 - Low/Low (negative values and negative means) corresponding to areas of lower priority, and Quadrants 3 - High/Low (positive values and negative means) and 4 - Low/High (negative values and positive means) corresponding to areas of intermediate priority.

### Ethical aspects

The information in this study was obtained from the National Public Health Surveillance System (Sivigila) of the Ministry of Health of Colombia. The research does not pose any risk because it uses secondary data, with no patient being identified.

## RESULTS

**Figure 2:**
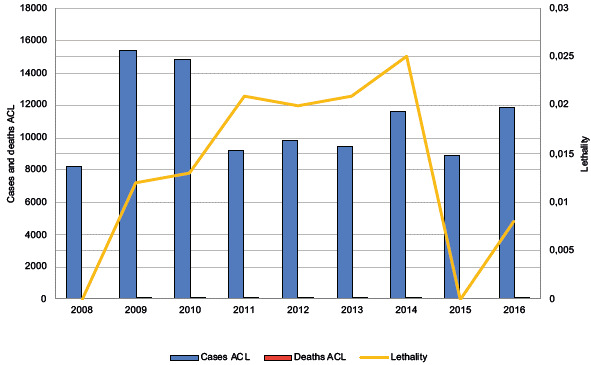
Temporal distribution of reported cases and deaths due to American cutaneous leishmaniasis in Colombia, 2008-2016

**Figure 3:**
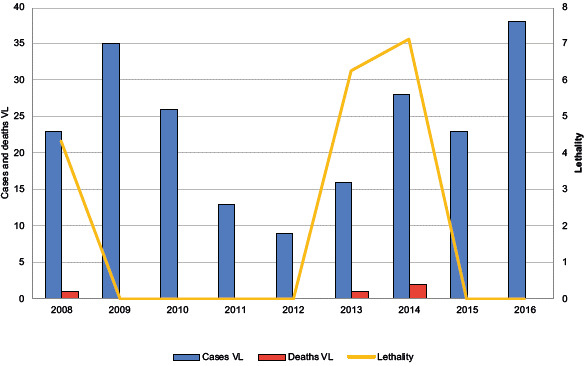
Temporal distribution of reported cases and deaths due to visceral leishmaniasis in Colombia, 2008-2016

In the study period, 99,503 cases of leishmaniasis were reported, of which 99,292 (99.78%) were ACL cases, with 14 deaths, which were associated with complications with treatment and 211 (0.21%) were VL cases, with four deaths. In 2009, the highest number of ACL cases was reported. In 2015, a low incidence was reported, and in the years 2008 and 2015, there were no deaths due to the disease. The distribution of cases and deaths from V was variable, with 2016 showing the highest number of reported cases in the series. The year 2012 showed the lowest lethality, and 2014, the highest (Figures 2 and 3).

Regarding the socio-demographic characteristics analyzed, it was necessary to exclude the cases that were self-referred and/or those classified as "other" for both ACL and VL. In all, 81,405 (82.11%) of the ACL cases were of male patients. Regarding age, 62,371 (62.91%) were aged between 21 and 50 years. Regarding ethnicity, the black/mulatto/Afro-Colombian group accounted for the highest proportion of cases, with 7,001 cases (65.92%), followed by the indigenous group, with 2,960 cases (27.87%). As for the housing area, 88.010 of the cases (88.63%) were reported in the rural area (Table 1).

**Table 1:** Sociodemographic characteristics of American cutaneous leishmaniasis cases in Colombia, 2008-2016

Variable	Frequency (N = 99,292)	Frequency Relative	%	CI of Relative Frequency
Sex (n = 99,135)				
Male	81,405	0.8	82.1	(0.821; 0.821)
Female	17,730	0.1	17.9	(0.178; 0.178)
Age (Categorized) (n=99113)				
<5	3,799	0.03	3.8	(0.038; 0.038)
5-9	4,445	0.04	4.5	(0.044; 0.044)
10-20	21,275	0.2	21.5	(0.214; 0.214)
21-50	62,371	0.6	62.9	(0.629; 0.629)
>50	7,223	0.07	7.3	(0.072; 0.072)
Ethnicity (n=10619)				
Indigenous	2,960	0.2	27.9	(0.278; 0.278)
ROM/Romani1	199	0.01	1.9	(0.018; 0.018)
Raizal2	401	0.03	3.8	(0.037; 0.037)
Palenquero3	58	0.005	0.5	(0.005; 0.005)
Black/mulatto/Afro-Colombian	7,001	0.6	65.9	(0.659; 0.659)
Living area				
Urban4	11,282	0.1	11.4	(0.113; 0.113)
Rural5	88,010	0.8	88.6	(0.886; 0.886)

CI = Confidence Interval. ^1^ROM = Romani Community; ^2^Raizal = Natives of San Andrés Archipelago, Providencia and Santa Catalina; ^3^Palenquero = People who inhabit the north coast; ^4^Urban = Geographical area that is defined by an urban perimeter; ^5^Rural = Populated center: centers that have a concentration of at least 20 adjoining houses located in the rural area, dispersed: dispersed houses and farms. DANE (2018); n = number of cases; % = percentage.

In all, 104 (52.52%) VL patients were male. As for the age group, 176 (89.34%) patients were under 5 years old. Regarding ethnicity, the indigenous group accounted for the highest proportion, with 37 cases (80.43%), followed by the black/mulatto/Afro-Colombian group with seven cases (15.21%). As for the housing area, 160 cases (75.82%) were reported in the rural area (Table 2).

**Table 2:** Sociodemographic characteristics of visceral leishmaniasis cases in Colombia, 2008-2016

Variable	Frequency (n total = 211)	Relative Frequency	%	CI of Relative Frequency
Sex (n = 198)				
Male	104	0,5	52.5	(0.528; 0.521)
Female	94	0,4	47.5	(0.478; 0.471)
Age (Categorized) (n = 197)				
<5	176	0.8	89.3	(0.894; 0.892)
5-9	8	0.04	4.1	(0.042; 0.038)
10-20	5	0.02	2.5	(0.026; 0.023)
21-50	5	0.02	2.5	(0.026; 0.023)
>50	3	0.01	1.5	(0.016; 0.013)
Ethnicity (n = 46)				
Indigenous	37	0.8	80.4	(0.811; 0.796)
ROM/Romani1	2	0.04	4.3	(0.051; 0.034)
Black/mulatto/Afro-Colombian	7	0.1	15.2	(0.166; 0.138)
Living area				
Urban2	51	0.2	24.2	(0.245; 0.238)
Rural3	160	0.7	75.8	(0.760; 0.756)

CI= Confidence Interval. ^1^ROM= Romani Community; ^2^Urban= Geographical area that is defined by an urban perimeter; ^3^Rural= Populated center: population centers that have a concentration of at least 20 continuous neighboring houses located in the rural area, dispersed: dispersed villas and farms. DANE (2018); n= number of cases; %= percentage.

The results of the logistic regression model of risk factors for VL in relation to ACL are presented below in Table 3.

**Table 3:** Summary of the Anal model of factors associated with visceral leishmaniasis in relation to American cutaneous leishmaniasis in Colombia, 2008-2016

	Estimate	OR	IC OR 95%	Pr(>|z|)
Intercept	-7.19	0.00	0.00 - 0.00	0.0000
Less than 5 years	3.22	25.12	13.20 - 55.76	0.0000
5-9 years	Ref.	1.00	NA	NA
10-20 years	-2.11	0.12	0.00 - 0.36	0.0002
21-50 years	-3.17	0.04	0.01 - 0.12	0.0000
Over 50 years	-1.66	0.18	0.04 - 0.06	0.0140
Rural area	Ref.	1.00	NA	NA
Urban area	1.50	4.50	3.14 - 6.36	0.0000
Afro-Colombian ethnicity	Ref.	1.00	NA	NA
Romani ethnicity	2.28	9.78	1.36 - 45.72	0.0075
Indigenous ethnicity	1.16	3.21	1.48 - 8.04	0.0059
Other ethnic groups	0.62	1.86	0.92 - 4.44	0.1128
Palenquero ethnicity	-11.42	0.00	0.00 - 0.00	0.9924
Raizal ethnicity	-12.20	0.00	0.00 - 0.00	0.9781

In the final model, sex, with significance of p<0,05, was not a significant variable.

In relation to the housing area, those living in urban areas had a 4.5-fold higher risk of contracting VL than ACL, indicating an urban pattern of VL in Colombia. Romani and indigenous ethnic groups had 9.78 and 3.21-fold increased risk, respectively, of contracting VL.

Regarding age, the age group at high risk of ACL was the "less than 5 years old" group (OR=25.12), while being aged between 21 and 50 years (OR=0.04) was considered a protective factor against ACL.

**Figure 4:**
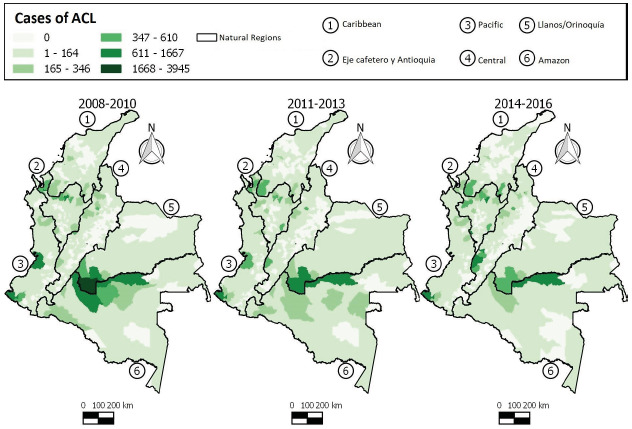
Distribution of American cutaneous leishmaniasis cases in Colombia, 2008-2016

ACL is widely distributed in Colombian territory, as seen in Figure 4. Many municipalities experienced a gradual increase in the number of cases during each 3-year period (2008-2010, 2011-2013, 2014-2016), strongly evident in the Caribbean region, Santa Rosa del Sur (Bolívar), Tierralta (Córdoba), Central region, Chaparral (Tolima), Ortega (Tolima), Planadas (Tolima), Rioblanco (Tolima), Rovira (Tolima), San Antonio (Tolima), Eje Cafetero y Antioquia region, Tarazá (Antioquia), Samaná (Caldas), and Pueblo Rico (Risaralda).

The municipalities of the Caribbean region, Valencia (Córdoba), Eje Cafetero y Antioquia, Santafé de Antioquia (Antioquia), Dabeiba (Antioquia), Necoclí (Antioquia), Valdivia (Antioquia), Pacífica region, Tadó (Chocó) and Amazon region, Florencia (Caquetá), and Mitú (Vaupés) showed fluctuations in the number of cases during the 9 years of study.

The municipalities of Eje Cafetero y Antioquia region, Apartado (Antioquia), Carepa (Antioquia), Turbo (Antioquia), Pacífica region, Buenaventura (Valle del Cauca), San andres de Tumaco (Nariño), Llanos/ Orinoquía region, La Macarena (Meta), Uribe (Meta), Vistahermosa (Meta), and Amazon region, San Vicente del Caguán (Caquetá) showed a reduction in the number of cases over the study period.

**Figure 5:**
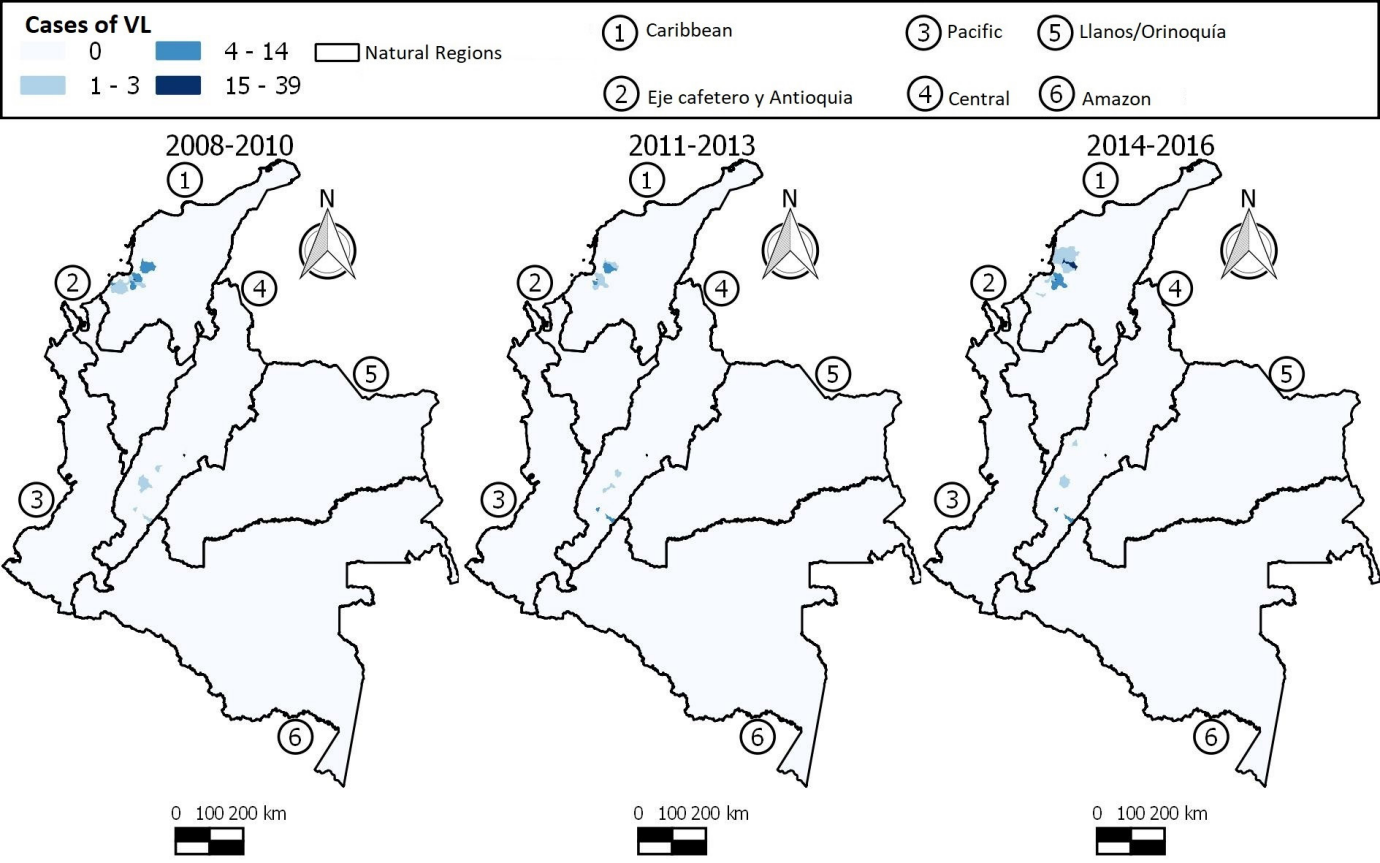
Distribution of visceral leishmaniasis cases in Colombia, 2008-2016

During the entire study period, the majority of the population affected by VL resided in the northern (the Caribbean) and central parts of the country (Figure 5). These regions saw an increase in the number of cases in the last 3-year period (2014-2016), especially in the municipalities of El Carmen de Bolívar (Bolívar), Maria La Baja (Bolívar), San Jacinto (Bolívar), San Juan Nepomuceno (Bolívar), Cereté (Córdoba), Purísima (Córdoba), San Onofre (Sucre), and Líbano (Tolima).

The municipalities of San Andrés Sotavento (Córdoba), Tuchín (Córdoba), Neiva (Huila), Sincelejo (Sucre), Corozal (Sucre), Palmito (Sucre), Sampués (Sucre), and Ortega (Tolima) presented fluctuations in the number of VL cases during all the trienniums, while the municipalities of Los Palmitos (Sucre), Ovejas (Sucre), and Coyaima (Tolima) showed a decline in the number of VL cases over the 3-year periods.

Positive and statistically significant values were observed in the trienniums 2008-2010 (0,341), 2011-2013 (0,346), and 2014-2016 (0,271), indicating a positive spatial autocorrelation and spatial dependence of ACL occurrence in Colombia as per the Moran Global Index.

**Figure 6:**
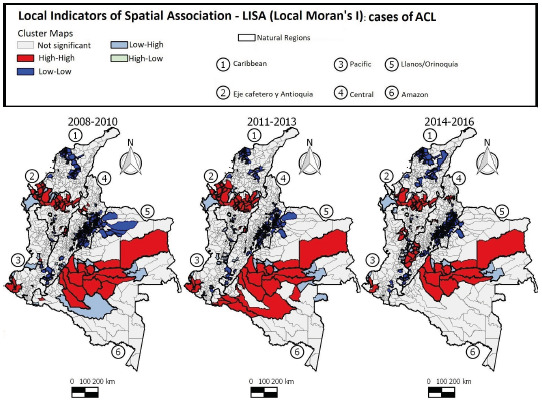
Distribution of American cutaneous leishmaniasis cases according to LISA standard of clusters in Colombia, 2008-2016

The LISA maps (Figure 6) show that local clusters of high incidence of ACL (High-High) in the first 3-year period were found in 50 municipalities located mainly in the Eje Cafetro y Antioquia, Llanos/Orinoquía, and Amazon regions. In the second 3-year period, 59 municipalities had similar pattern (High/High), with changes in the dispersion in the Amazon region. In the third 3-year period, 51 municipalities were identified as having a positive association (High-High), with the clusters decreasing in the Eje Cafetro y Antioquia and Amazon regions and dispersing to the Central region, indicating that the pattern of ACL occurrence in Colombia is not static and the disease may occasionally spread to other areas of the country.

Positive and statistically significant values were observed in the trienniums 2008-2010 (0,284), 2011-2013 (0,100), and 2014-2016 (0,096), indicating a positive spatial autocorrelation and spatial dependence of the occurrence of VL in Colombia by the Moran Global Index.

**Figure 7:**
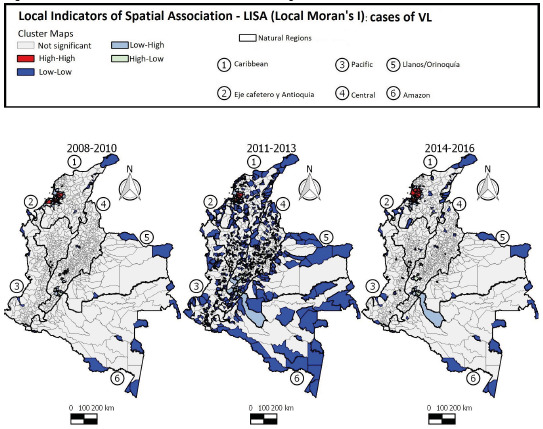
Distribution of visceral leishmaniasis cases according to LISA standard of Clusters in Colombia, 2008-2016

The LISA cluster maps (Figure 7) showed that during the first 3 years, 14 municipalities presented the High-High pattern for VL. In the second 3-year period, seven municipalities showed the same patterns (positive values and positive means). In the following 3 years, 13 municipalities were highlighted as High-High clusters. During the entire study period, the local clusters with high incidence of VL were found mainly in the Caribbean region, showing dispersion of the disease with an increase in the number of cases from the second to the third triennium.

## DISCUSSION

Leishmaniasis are complex and multifactorial in terms of time, space and determinants. Cultural, environ-mental, socioeconomic, and political reasons can play an important role in the emergence and dispersion of new cases. The constant displacement of the Colombian population due to the violence and political instability experienced during the study period may have strongly affect the dispersion of vectors, reservoirs, and consequently the disease-causing pathogens, favoring their distribution throughout the country, as described by González 17, Ferro 18, Berry and Berrang-Ford 19, Ramírez 20, González 21, Patino 22, Herrera 23, Hernández 24, and Ovalle-Bracho 25. Thus, the hosts may be prone to increased risks of infection, since several ecological and social determinants coexist in the environment 26.

Our study presents important information on the epidemiological situation and the distribution of leishmaniasis in Colombia in recent years. In this country, leishmaniasis not only showed an increase in the number of cases during the study period but also a variation in the transmission cycle and spatial distribution with interannual changes. The reasons for this increase are unknown but could be explained by improved clinical and laboratory diagnosis methods and surveillance systems and climate change 18,27.

During the first and last 3-year periods, a large number of cases and high incidences of both ACL and VL were observed, possibly explained by the climatic variation caused by the El Niño, which was intense during 2014-2016. It can also be explained by the outbreak of CL that occurred during 2005-2009 among soldiers and dogs of the Colombian army in the Pacific, Llanos/Orinoquía, and Amazon regions during the armed conflict in the country in this period 28 and by the outbreak that occurred during the years 2003-2007 in the municipality of Chaparral (Tolima) in the Central region 29.

According to the last survey conducted by the DANE, in the year 2018, approximately 23% of the general population lived in rural areas, and in the present study, the occurrence of most cases of both ACL and VL in these places was reported. Perhaps, people who work in rural areas, such as farmers and traders, who according to the Fedesarrollo 30 are the main workers in the rural sector, and army soldiers, who must work in both rural and urban areas, are highly exposed to the disease 21,22,28. It is important to note that there is a wide variety of hosts in rural areas and this increases the risk of contracting infection in the rural population.

In Colombia, most of the current studies are about ACL. In the present study, this disease accounted for the majority of reported cases of leishmaniasis in 2008-2016, with a high percentage being reported in Bogotá (which is not considered an area of high ACL risk because patients from other nearby areas visit this place for treatment), Antioquia, and Tolima, and a high number of cases in Eje Cafetro y Antioquia, Llanos/Orinoquía, and Amazon, corroborating the results presented by Herrera 23. According to PAHO/WHO 31, during the period of 2016-2018, San Andres de Tumaco (Nariño) and Rovira (Tolima), belonging to the Pacific and Central regions, respectively, were among the 10 second-level administrative units that presented the highest averages of LC cases in the Americas.

The population affected by ACL in Colombia showed male predominance, and the majority was aged between 21 and 50 years, had laboratory-confirmed diagnosis, was not hospitalized, and subsequently cured. All this information is in line with the data presented in the epidemiological report of the Americas 2016, which contained no information on the outcomes of the patients. In the state of Alagoas, from 1999 to 2008, males aged over 10 years were frequently affected by ACL 32; in Peru and Ecuador, those aged 0-10 years were frequently affected 33.

Regarding VL, most of the cases were reported among males aged below 5 years of age; most patients had laboratory-confirmed diagnosis, were hospitalized, and were cured. Similar results were found in Belo Horizonte and Alagoas, Brazil, with the largest portion of patients being male and aged between 0 and 11 years, and most patients were cured 11,34. An outbreak that occurred in the urban area of Neiva (Huila) located in Central region of Colombia in the year 2012, also affected minors under 5 years, with a predominance of female sex; most patients had laboratory-confirmed diagnosis, were hospitalized, and were cured 6.

In the studied period, the Caribbean region had the highest occurrence of VL cases, in line with the findings of Paternina Tuirán 7, Herrera 8, and Rivero-Rodríguez 10, who found a high occurrence of cases in humans and dogs in this region.

Leishmaniasis can occur concomitantly with other diseases, and the human immunodeficiency virus (HIV) is most common virus in cases of co-infection 11,35,36. This characteristic makes the prognosis of leishmaniasis complex, as comorbidities can alter the pathogenicity of the disease to the host 37. As a limitation, a large percentage of notification forms did not contain data on HIV co-infection, which led to an incomplete analysis of the situation for this population considered at risk and understanding its epidemiology, clinical manifestations, severity, and evolution. Although leishmaniasis must compulsorily be reported, underreporting still occurs, which is one of the limitations of this study. In order to minimize such limitations, the analyses considered the estimates of neighboring municipalities to reduce the risk of underestimation since the levels of underreporting may be different in the different municipalities.

Early diagnosis can prevent the occurrence of disabilities and death 38; vector control can contribute to the reduction of disease transmission 3, and reporting of cases to the health system can help provide accurate information 25.

Our results highlight a high risk of ACL in 29 municipalities (2.58%) and of VL in eight municipalities (0.71%), which should be prioritized in terms of surveillance and disease control measures. The proposal presented here helps identify areas that need prioritization and a combination of strategies to address the risk of ACL and VL in order to improve the effectiveness of disease control measures.

We found that the leishmaniasis notification forms had some missing data, and this can make data analysis difficult. This fact demonstrates the need for training of professionals involved in filling out the disease-notification forms. It is important to take action to prevent and control the occurrence of leishmaniasis throughout the Colombian territory, focusing on the Caribbean, Central, and Eje Cafetero y Antioquia regions, which had the highest number of cases during the study period. However, it is necessary to identify the main risk factors in these localities in order to be able to implement measures that are adapted to specific local contexts. The results of this study reflect the epidemiological situation and the distribution of leishmaniasis in recent years in Colombia. These data can be used as evidence for the implementation of public prevention and control policies in the country ♣
